# Adenovirus DNA in Guthrie cards from children who develop acute lymphoblastic leukaemia (ALL)

**DOI:** 10.1038/sj.bjc.6605581

**Published:** 2010-03-02

**Authors:** E Honkaniemi, G Talekar, W Huang, G Bogdanovic, E Forestier, U von Doblen, M Engvall, D A Ornelles, L R Gooding, B Gustafsson

**Affiliations:** 1 Department of Clinical Science, Intervention and Technology, Astrid Lindgren Children's Hospital, Karolinska University Hospital-Huddinge, CLINTEC, Karolinska Institutet, Stockholm S-141 86, Sweden; 2 Department of Microbiology and Immunology, Emory University School of Medicine, Atlanta, GA, USA; 3 Department of Clinical Microbiology, Karolinska University Hospital, Stockholm, Sweden; 4 Department of Microbiology, Tumour and Cell Biology, Karolinska Institutet, Stockholm, Sweden; 5 Department of Clinical Science, Paediatrics, Umeå University Hospital, Umeå, Sweden; 6 Department of Laboratory Medicine, Division of Metabolic Diseases, Karolinska University Hospital Huddinge, Karolinska Institutet, Stockholm, Sweden; 7 Karolinska Institutet, Stockholm, Sweden; 8 Department of Microbiology and Immunology, Wake Forest University School of Medicine, Winston-Salem, NC, USA

**Keywords:** aetiology, childhood leukaemia, adenovirus, prenatal infection

## Abstract

**Background:**

In search of a proposed viral aetiology of childhood acute lymphoblastic leukaemia (ALL), the common species C adenoviruses were analysed in Guthrie cards.

**Methods:**

Guthrie cards from 243 children who later developed ALL and from 486 matched controls were collected and analysed by nested polymerase chain reaction for the presence of adenovirus DNA.

**Results:**

Adenovirus DNA was reliably detected from only two subjects, both of whom developed ALL.

**Conclusion:**

Adenovirus DNA is detected in Guthrie card samples at too low a frequency to reveal an association between adenovirus and the development of leukaemia.

Acute lymphoblastic leukaemia (ALL) is the most common malignancy in childhood, with an annual incidence of three to four cases per 100 000 children in developed countries. Although the aetiology in over 95% of ALL is unknown (Doll, 1989; Margolin, 1997), it is widely believed that an infectious agent may have an effect in the development of this haematological malignancy. We recently reported that adenovirus DNA was detected in 13 of 51 Guthrie cards from ALL patients, but in only 6 of 47 healthy controls (*P*=0.0122, Fisher's exact test), indicating that adenovirus may be such a causative agent (Gustafsson *et al*, 2007). However, a subsequent study of twice as many paediatric ALL patients in California failed to find adenovirus DNA in the corresponding neonatal blood spots (Vasconcelos *et al*, 2008). This study sought to further examine the possibility of an association between prenatal adenovirus infection and ALL using a larger number of archived Swedish samples from children who later developed ALL and controls without the disease.

## Materials and methods

The patient population included 243 Swedish children who were diagnosed with ALL between 1992 and 2006. The mean age was 5.4 years and the median was 4.3 years (range 67 days to 15 years). Patients were randomly selected from the Nordic Society of Paediatric Haematology and Oncology register. Ten of these 243 children were included in our previous study (Gustafsson *et al*, 2007) and two of them were positive for adenovirus DNA in that study. More than half (58%) of the subjects were male and 217 (89%) of the leukaemias were of B-cell lineage. As controls, Guthrie cards from 486 children with no known history of leukaemia were picked two Guthrie cards apart from the patient Guthrie card in the Guthrie card archive, thereby matching for birthplace and birthdate.

The blood from infants that was collected on Guthrie cards at 3–5 days of age was processed as described previously (Barbi *et al*, 1996; Priftakis *et al*, 2003), except that a total of four punches were harvested from each card compared with three punches in the previous study. This study was approved by the Ethics Committee at Karolinska Institutet and by the Institutional Review Board of Emory University.

The material recovered from four 3-mm punches from a Guthrie card represented approximately 12 *μ*l of blood containing about 180 000 leukocytes and 120 000 lymphocytes in a final volume of 60–80 *μ*l. Samples were first tested for the presence of DNA by real-time polymerase chain reaction (PCR) for the human albumin gene (Laurendeau *et al*, 1999; Desire *et al*, 2001), using 5 *μ*l of material from each sample. The sensitivity of the method was 10 copies per reaction. Positive samples were coded and sent to Atlanta, where they were screened for the presence of species C adenovirus DNA by nested PCR (nPCR-2 in Garnett *et al*, 2009) using primers P11–P14, which were designed to amplify a conserved region of the species C adenovirus hexon gene corresponding to nucleotides 18 838–19 205 of Ad2 (GenBank accession no. BK000407). Samples having one or more positive reactions with nPCR-2 were then re-tested in duplicate with nPCR-2 and with another nested PCR assay (nPCR-1 in Garnett *et al*, 2009) using primers P7–P10 derived from the Ad2 hexon sequence, to amplify nucleotides 20 721–21 572. Both adenovirus-specific PCR assays routinely detect fewer than five copies of the viral genome. Products of nPCR-1 from samples testing positive by both reactions were sequenced at Eurofins MWG Operon (Huntsville, AL, USA) to confirm the adenovirus serotype. The sequences were aligned by Lasergene software (DNAStar, Inc., Madison, WI, USA) and compared with the sequences from species C adenoviruses in GenBank.

## Results

DNA was successfully extracted from the Guthrie cards of 243 patients and from 484 of 486 controls. Two of the control samples were negative for the albumin gene by real-time PCR and were therefore excluded. All samples were analysed in duplicate reactions for the presence of species C adenovirus DNA using hexon-specific nPCR-2 (Garnett *et al*, 2009). Only nine of the 727 samples yielded a positive result in at least one PCR reaction. These nine samples were re-tested using both nPCR-2 and nPCR-1, which detected a different region of the hexon gene (Garnett *et al*, 2009). Two of the nine samples were confirmed to contain adenoviral DNA by their yielding at least one positive result in each pair of reactions performed with both hexon-specific PCR assays. The other seven samples failed to yield a positive reaction with either PCR assay and were considered negative. Both positive samples were derived from ALL donor Guthrie cards. Both positive samples were confirmed to contain Ad2 DNA by sequencing the product of nPCR-1 (Garnett *et al*, 2002, 2009).

## Discussion

The reasons for the discrepancy between this investigation and our previous preliminary study are unclear. Quantitative analysis for a single-copy cellular gene indicated that the DNA present in Guthrie card extractions in this study was significantly more concentrated than that used in the preliminary report (data not shown). Controls analysed in the previous study eliminated the likelihood of PCR procedural contamination. Nonetheless, levels of adenovirus DNA measured by quantitative real-time PCR showed very low levels of viral DNA in the positive samples. We are unable to exclude the possibility of random environmental contamination in the previous study, although no negative controls were found to contain adenoviral DNA.

The low fraction of Guthrie cards positive for adenoviral DNA is in sharp contrast with studies on amniotic fluid. Of 1187 unique amniocentesis samples analysed in four separate studies of sonographically normal pregnancies, more than 1 in 20 (5.4%) contained adenoviral DNA (Van den Veyver *et al*, 1998; Wenstrom *et al*, 1998; Baschat *et al*, 2003; Reddy *et al*, 2005). Remarkably, subsequent studies have detected no increased morbidity in infants following prenatal infection with adenovirus, as determined by PCR of amniocentesis samples (Miller *et al*, 2009), suggesting that prenatal infection, similar to postnatal infection, with species C adenoviruses is largely asymptomatic. Given a prenatal infection rate of 5%, our Guthrie card analysis seriously underestimates the frequency of fetal adenovirus infections, which is consistent with the biology of this pathogen. These results lead us to conclude that adenovirus DNA is not readily detected in the archived neonatal blood spots, irrespective of whether the subject subsequently develops leukaemia. Although the findings reported here fail to support our previously reported preliminary results (Gustafsson *et al*, 2007), these findings agree with those reported subsequently (Vasconcelos *et al*, 2008).

Adenovirus is not commonly found in peripheral blood, except during a fulminant infection (Flomenberg *et al*, 1997; Perlman *et al*, 2007). By contrast, a large majority of lymphocyte samples isolated from the adenoids or tonsils of normal children undergoing tonsillectomy or adenoidectomy contain adenovirus DNA (Garnett *et al*, 2002, 2009). Thus, after acute infection, adenovirus DNA may be restricted to mucosal lymphoid compartments in the infected neonate, as well as in the latently infected child, which may explain our inability to detect this virus in a high fraction of Guthrie cards containing peripheral blood.

Although these findings show no association between the development of leukaemia and adenovirus DNA in neonatal blood, this pathogen remains an intriguing candidate for a role in the aetiology of leukaemia. Species C adenovirus oncoproteins that disable the cellular DNA repair machinery (Weitzman and Ornelles, 2005) also have the power to transform cells by a ‘hit and run’ mechanism (Nevels *et al*, 2001; Endter and Dobner, 2004), making species C adenovirus a candidate for promoting the initial genetic lesion leading to leukaemia. Adenovirus may also have a role in the second hit postulated in the delayed infection model of leukaemia (Greaves, 2006). The fraction of children undergoing tonsillectomies with adenovirus DNA in their mucosal lymphocytes peaks between 2 to 5 years (see Figure 1 of Garnett *et al*, 2009), which coincides with the peak presentation of childhood ALL (Greaves, 2006).

## References

[bib1] Barbi M, Binda S, Primache V, Luraschi C, Corbetta C (1996) Diagnosis of congenital cytomegalovirus infection by detection of viral DNA in dried blood spots. Clin Diagn Virol 6: 27–32 15566887 10.1016/0928-0197(96)00228-0

[bib2] Baschat AA, Towbin J, Bowles NE, Harman CR, Weiner CP (2003) Prevalence of viral DNA in amniotic fluid of low-risk pregnancies in the second trimester. J Matern Fetal Neonatal Med 13: 381–384 12962262 10.1080/jmf.13.6.381.384

[bib3] Desire N, Dehee A, Schneider V, Jacomet C, Goujon C, Girard PM, Rozenbaum W, Nicolas JC (2001) Quantification of human immunodeficiency virus type 1 proviral load by a TaqMan real-time PCR assay. J Clin Microbiol 39: 1303–1310 11283046 10.1128/JCM.39.4.1303-1310.2001 PMC87929

[bib4] Doll R (1989) The epidemiology of childhood leukemia. J R Stat Soc Ser Series A 152: 341–351

[bib5] Endter C, Dobner T (2004) Cell transformation by human adenoviruses. Curr Top Microbiol Immunol 273: 163–214 14674602 10.1007/978-3-662-05599-1_6

[bib6] Flomenberg P, Gutierrez E, Piaskowski V, Casper JT (1997) Detection of adenovirus DNA in peripheral blood mononuclear cells by polymerase chain reaction assay. J Med Virol 51: 182–188 9139081 10.1002/(sici)1096-9071(199703)51:3<182::aid-jmv7>3.0.co;2-2

[bib7] Garnett CT, Erdman D, Xu W, Gooding LR (2002) Prevalence and quantitation of species C adenovirus DNA in human mucosal lymphocytes. J Virol 76: 10608–10616 12368303 10.1128/JVI.76.21.10608-10616.2002 PMC136639

[bib8] Garnett CT, Talekar G, Mahr JA, Huang W, Zhang Y, Ornelles DA, Gooding LR (2009) Latent species C adenoviruses in human tonsil tissues. J Virol 83: 2417–2428 19109384 10.1128/JVI.02392-08 PMC2648257

[bib9] Greaves M (2006) Infection, immune responses and the aetiology of childhood leukaemia. Nat Rev 6: 193–203 10.1038/nrc1816 16467884

[bib10] Gustafsson B, Huang W, Bogdanovic G, Gauffin F, Nordgren A, Talekar G, Ornelles DA, Gooding LR (2007) Adenovirus DNA is detected at increased frequency in Guthrie cards from children who develop acute lymphoblastic leukaemia. Br J Cancer 97: 992–994 17876329 10.1038/sj.bjc.6603983 PMC2360426

[bib11] Laurendeau I, Bahuau M, Vodovar N, Larramendy C, Olivi M, Bieche I, Vidaud M, Vidaud D (1999) TaqMan PCR-based gene dosage assay for predictive testing in individuals from a cancer family with INK4 locus haploinsufficiency. Clin Chem 45: 982–986 10388473

[bib12] Margolin J (1997) Acute Lymphoblastic Leukemia. Lippincott-Raven: New York

[bib13] Miller JL, Harman C, Weiner C, Baschat AA (2009) Perinatal outcomes after second trimester detection of amniotic fluid viral genome in asymptomatic patients. J Perinatal Med 37: 140–143 10.1515/JPM.2009.027 18956964

[bib14] Nevels M, Tauber B, Spruss T, Wolf H, Dobner T (2001) ‘Hit-and-run’ transformation by adenovirus oncogenes. J Virol 75: 3089–3094 11238835 10.1128/JVI.75.7.3089-3094.2001 PMC114102

[bib15] Perlman J, Gibson C, Pounds SB, Gu Z, Bankowski MJ, Hayden RT (2007) Quantitative real-time PCR detection of adenovirus in clinical blood specimens: a comparison of plasma, whole blood and peripheral blood mononuclear cells. J Clin Virol 40: 295–300 17959413 10.1016/j.jcv.2007.09.003

[bib16] Priftakis P, Dalianis T, Carstensen J, Samuelsson U, Lewensohn-Fuchs I, Bogdanovic G, Winiarski J, Gustafsson B (2003) Human polyomavirus DNA is not detected in Guthrie cards (dried blood spots) from children who developed acute lymphoblastic leukemia. Med Pediatr Oncol 40: 219–223 12555248 10.1002/mpo.10246

[bib17] Reddy UM, Baschat AA, Zlatnik MG, Towbin JA, Harman CR, Weiner CP (2005) Detection of viral deoxyribonucleic acid in amniotic fluid: association with fetal malformation and pregnancy abnormalities. Fetal Diagn Ther 20: 203–207 15824499 10.1159/000083906

[bib18] Van den Veyver IB, Ni J, Bowles N, Carpenter Jr RJ, Weiner CP, Yankowitz J, Moise Jr KJ, Henderson J, Towbin JA (1998) Detection of intrauterine viral infection using the polymerase chain reaction. Mol Genet Metab 63: 85–95 9562961 10.1006/mgme.1997.2651

[bib19] Vasconcelos GM, Kang M, Pombo-de-Oliveira MS, Schiffman JD, Lorey F, Buffler P, Wiemels JL (2008) Adenovirus detection in Guthrie cards from paediatric leukaemia cases and controls. Br J Cancer 99: 1668–1672 19002185 10.1038/sj.bjc.6604714 PMC2584954

[bib20] Weitzman MD, Ornelles DA (2005) Inactivating intracellular antiviral responses during adenovirus infection. Oncogene 24: 7686–7696 16299529 10.1038/sj.onc.1209063

[bib21] Wenstrom KD, Andrews WW, Bowles NE, Towbin JA, Hauth JC, Goldenberg RL (1998) Intrauterine viral infection at the time of second trimester genetic amniocentesis. Obstetr Gynecol 92: 420–424 10.1016/s0029-7844(98)00210-5 9721782

